# Primary closure of a spontaneous esophageal rupture under hand-assisted laparoscopy: a case report

**DOI:** 10.1186/s40792-016-0204-z

**Published:** 2016-07-23

**Authors:** Ryuichi Mikami, Yoshihiko Nakamoto, Hirokuni Ikeda, Hiroyuki kayata, Teppei Murakami, Mitsuo Yamamoto

**Affiliations:** Department of Surgery, Kobe City Medical Center West Hospital, 4-2 Ichibancho, Nagata-ku, Kobe City, Hyogo 653-0013 Japan

**Keywords:** Boerhaave’s syndrome, Laparoscopy, Primary repair

## Abstract

Spontaneous rupture of the esophagus, which is also known as Boerhaave’s syndrome, is a rare life-threatening condition that requires urgent surgical management. The optimal treatment involves surgical repair of the esophageal defect, which is usually accomplished via laparotomy, thoracotomy, or both, and mediastinal debridement. Here, we report a case of spontaneous rupture of the esophagus that was treated with suturing repair and drain insertion using a hand-assisted laparoscopic approach.

## Background

Spontaneous rupture of the esophagus is an emergency condition in which all layers of the esophageal wall are perforated due to a sharp increase in esophageal pressure (e.g., due to vomiting). It can be fatal if it is not treated appropriately at an early stage [1, 2]. Conservative treatment might be indicated if the rupture is small, any contamination is mild, and the patient’s physical status remains good, but surgery is the standard treatment. We report a case that was treated with simple suture closure under hand-assisted laparoscopy, which resulted in a favorable outcome.

## Case presentation

**Fig. 1:**
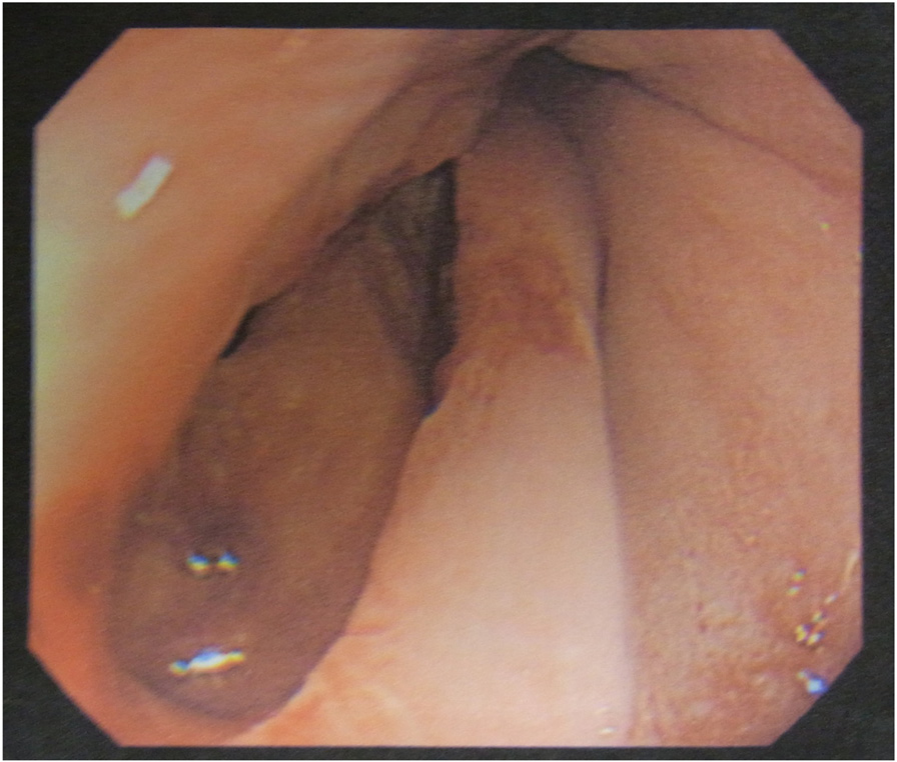
Upper gastrointestinal endoscopy revealed a left-sided rupture of the lower esophagus

**Fig. 2:**
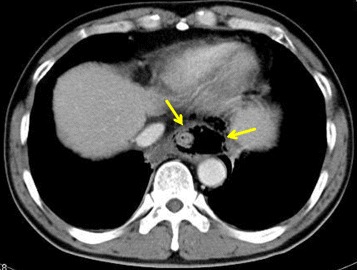
A chest CT scan revealed pneumomediastinum

**Fig. 3:**
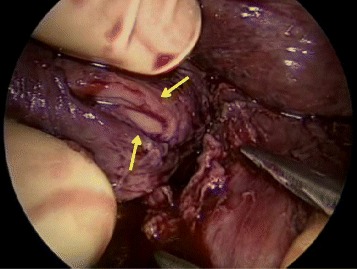
The perforation site. A longitudinal perforation measuring approximately 2 cm was clearly visible on the left side of the esophagus

A 61-year-old male with a history of alcoholism and open cholecystectomy was admitted to our hospital for hematemesis. On examination, he was tachycardic and tachypneic. His blood test results were as follows: total white blood cell count 16.1 × 10^3^/L, platelet count 9.3 × 10^4^/L, C-reactive protein level 10.3 mg/dL, blood urea nitrogen level 30 mg/dL, serum creatinine level 1.37 mg/dL, and endotoxin level 7.5 pg/mL. Upper gastrointestinal endoscopy revealed a left-sided rupture of the lower esophagus (Fig. 1), and a chest CT scan revealed pneumomediastinum (Fig. 2), which was suggestive of the intra-mediastinal type of Boerhaave’s syndrome. The patient underwent emergency surgery 24 h after the onset of his symptoms. A 7-cm upper midline incision was made through the skin for the hand-assisted laparoscopy technique. Two 5-mm ports were produced in the left hypochondrium and left flank. A 12-mm port for a video camera was made in the left upper quadrant. Under hand-assisted laparoscopy, while expanding a field of vision of the esophageal hiatus, the abdominal esophagus was encircled and the left diaphragmatic crus was dissected with ultrasonically activated scalpel. Subsequently, intraoperative endoscopy was performed; we identified the site of the esophageal rupture at the same level as diaphragm. A longitudinal perforation of the left side of the esophagus measuring approximately 2 cm was clearly visible (Fig. 3). A cloudy fluid collection with gastric juice and blood clots was seen around the site of perforation in the mediastinum.

**Fig. 4:**
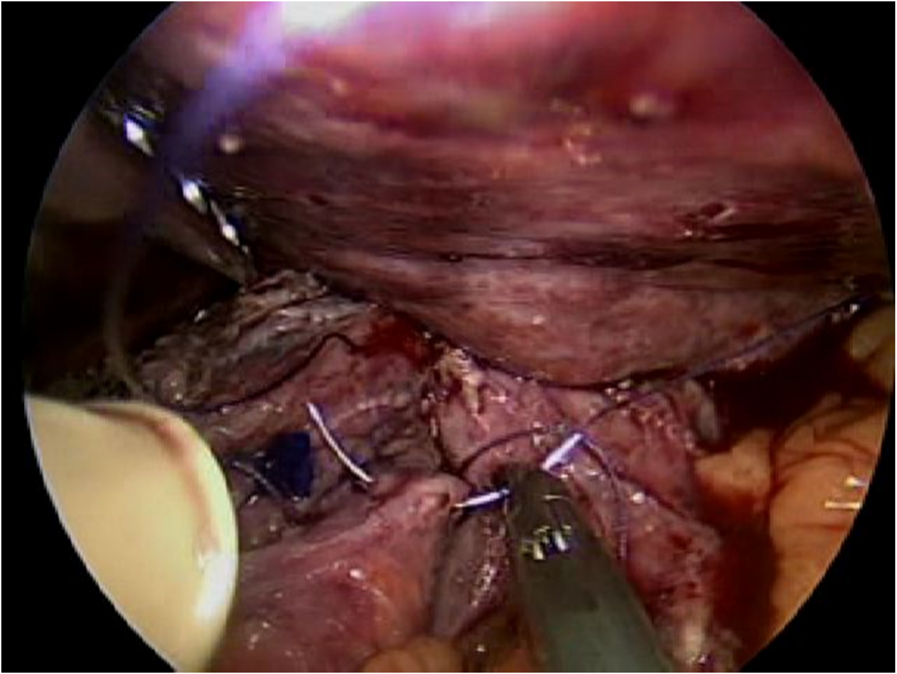
Repair of the perforation. The ruptured site was closed with a two-layered suture under hand-assisted laparoscopy

The associated crushing injury was mild, and the rupture site was closed with a two-layered suture under hand-assisted laparoscopy (Fig. 4). The closed site was not covered by the omentum or any other tissue. A leakage test was performed. Ultimately, a drain was placed in the posterior mediastinum, and a feeding jejunostomy was constructed. The patient recovered without any complications. He was transferred from the intensive care unit to a general ward in their progress from being critically ill to recovering. A diatrizoate meglumine esophagogram obtained at 9 days after the operation did not show any extravasation. He was discharged from hospital at 3 weeks after the operation.

### Discussion

Boerhaave’s syndrome involves the spontaneous transmural rupture of the esophagus and was first described in 1724 by Dr. Herman Boerhaave [3]. Despite considerable improvements in diagnostic, therapeutic, and intensive care techniques, esophageal perforation remains a life-threatening condition and is associated with high morbidity and mortality rates (ranging from 10 to 50 %) [4]. It is considered to be caused by a rapid increase in pressure within the esophagus, e.g., during vomiting, together with the failure of the cricopharyngeus muscle to relax. Such ruptures often occur at sites of anatomical weakness, e.g., the part of the left posterolateral wall of the lower third esophagus located 2 to 3 cm from the gastroesophageal junction [5].

In cases of esophageal rupture, the most important aim of any treatment should be immediate closure of the esophageal tear. There have been several reports about cases in which Boerhaave’s syndrome was successfully managed with a self-expandable metallic stent [6–10]. Recently, endoscopic closure using a clipping device has also been reported [11–13], but surgical treatment is usually selected, as advanced mediastinal intrapleural contamination by the contents of the gastrointestinal tract is often seen at the initial examination. Regarding surgery for spontaneous esophageal rupture, previous reports have suggested that most procedures are performed via a thoracotomy or laparotomy, and there have been few reports about camera-assisted operations being performed for spontaneous esophageal rupture.

A search of PubMed using the keywords “thoracoscopy,” “laparoscopy,” and “Boerhaave’s syndrome” revealed that camera-assisted operations have been performed for spontaneous esophageal rupture in seven cases [14–20], and simple suture closure of the perforated site under thoracoscopy or laparoscopy was carried out in six of these cases [14–18, 20]. Ashrafi et al. state that indications for the minimally invasive technique for spontaneous esophageal rupture are limited to patients with stable hemodynamics, without signs of escalating sepsis, and without concomitant conditions contraindicated for surgeries, including laparoscopic or thoracoscopic surgery [16]. However, Vaidya et al. reported that they selected a thoracoscopic approach for a patient with septic shock [19]. Thus, at present, there are no clear strategies for the selection of endoscopic treatment. Previous studies, including ours, reported that the perforations were 2–3 cm in diameter, suggesting that endoscopic surgery is well indicated for patients in whom the perforation site is small, but that, in patients in whom the perforation site is so large that it must be closed with sutures and covered by the omentum or any other tissue, open-chest or abdominal surgery needs to be performed to secure sutures without sticking to endoscopic surgery. The advantages of endoscopic surgery include the following: (1) it is minimally invasive because it minimizes the injury to the chest and abdominal walls; (2) it carries a low risk of wound infection; and (3) it provides a wider surgical field. On the other hand, its disadvantages are as follows: (1) the operative time is prolonged; (2) the removal of contaminants may be insufficient; and (3) the surgeon needs to be skillful in the manipulation of sutures.

As for the approach used to gain access to the mediastinum, the transabdominal approach is considered to be more useful than the transthoracic approach as it causes less operative stress and allows a drain to be inserted into the appropriate part of the posterior mediastinum, the use of the greater omentum, and the addition of an intestinal fistula. In the present case, as the perforation did not open into the thorax, we selected the transesophageal approach. Patients with esophageal perforations are often in a poor physical condition, and camera-assisted operations, which take a considerable amount of time, are not suitable for such cases. However, at our hospital, camera-assisted approaches have been selected for standard esophageal and gastric operations since 2003. In the current case, we selected a laparoscopic approach, even though the patient required an emergency operation, based on the assumption that the enlarged view that this would provide would allow us to definitively identify and close the perforation site. In addition, since the patient had a history of cirrhosis, and preoperative CT revealed marked enlargement of the left hepatic lobe, we predicted inadequate widening of the surgical field at the esophageal hiatus under complete endoscopic visualization and performed hand-assisted laparoscopic surgery. Successful lifting of the lateral hepatic segment and traction of the stomach by the surgeon’s left hand enabled us to close the perforation in a wide surgical field.

Concerning the closure of the perforation site, it has been reported that dysraphia is more likely to occur in cases in which 24 h or more have passed since the onset of the condition [4], but as the enlarged view provided by the laparoscope revealed that the perforation site was small in diameter and did not exhibit necrosis or severe crushing, we performed simple closure under hand-assisted laparoscopy, and the patient’s postoperative course was favorable.

It is not possible to conduct a comparative study of the various methods that can be used to treat spontaneous esophageal rupture in cases that require emergency surgery, as the number of cases is limited. Therefore, the further accumulation of cases is necessary, but as camera-assisted operations are minimally invasive, such procedures should be considered under certain conditions (e.g., depending on the skill level of the surgeon and whether the institution has the appropriate facilities to perform such operations).

## Conclusions

While the drawbacks of camera-assisted surgery need to be carefully considered during treatment selection for spontaneous esophageal rupture, camera-assisted surgery should be considered as a potential treatment option as it is minimally invasive, and the enlarged view it provides allows the definitive repair of the perforation site.

## Consent

Written informed consent was obtained from the patient for publication of this case report and accompanying images. A copy of the written consent is available for review by the editor-in-chief of the journal.

## Abbreviation

CT, computed tomography
